# iTRAQ-Based Proteomic Profiling of Skin Aging Protective Effects of *Tremella fuciformis*-Derived Polysaccharides on D-Galactose-Induced Aging Mice

**DOI:** 10.3390/molecules29215191

**Published:** 2024-11-02

**Authors:** Yuanyuan Xu, Xiaofei Liu, Jingjing Guan, Jin Chen, Xiaofei Xu

**Affiliations:** 1 College of Food Science and Engineering, Guangdong Ocean University, Yangjiang 529500, China; 2 Yangjiang Institute of Guangdong Ocean University, Yangjiang 529500, China

**Keywords:** anti-skin aging, oxidative stress, inflammation, differentially expressed protein, mushroom polysaccharides

## Abstract

In the present study, a heteromannan primarily composed of mannose, fucose, xylose, glucose, and arabinose at a molar ratio of 4.78:1.18:1:0.82:0.11 containing a low proportion of glucuronic acid with weight-average molecular weights of 3.6 × 10^6^ Da, named NTP, was prepared from the fruiting body of *Tremella fuciformis*. The anti-skin-aging effects of NTP on d-Galactose-induced aging mice and the biological mechanisms were investigated by an iTRAQ-based proteomics approach. NTP substantially mitigated skin aging characterized by a decreased loss of hydroxyproline and hyaluronic acid and reduced oxidative stress in the skin. Moreover, 43 differentially expressed proteins (DEPs) were identified in response to NTP, of which 23 were up-regulated and 20 were down-regulated. Bioinformatics analysis revealed that these DEPs were mainly involved in the biological functions of cellular and metabolic regulations, immune system responses, and structural components. The findings provided new insights into the biological mechanisms underlying the anti-skin-aging actions of *T. fuciformis*-derived polysaccharides and facilitated NTP applications in naturally functional foods.

## 1. Introduction

Mushrooms are macrofungi containing a wide range of bioactive components and simultaneously have a low content of calories, cholesterol, and lipids [1]. Due to their multiple health-promoting effects and nutritional attributes, mushrooms are considered unlimited resources for nutraceuticals and functional foods [2]. The fruiting body of *Tremella fuciformis* Berk. (*T. fuciformis*), widely known as snow ear or silver ear, is an edible mushroom and has been consumed over hundreds of years as food and medicinal material in China because of its excellent nutritional and medical value [3]. The fruiting body of *T. fuciformis* is rich in polysaccharides. In recent decades, many studies have illustrated *T. fuciformis* polysaccharides (TPs) as the major active substances, which are associated with numerous bioactivities, including antitumor activity, immune modulation, antioxidation and anti-aging activity, hypoglycemic and hypolipidemic effects, neuroprotection, the promotion of wound healing, photoprotection, etc. [3,4,5]. To date, many types of TPs have been reported. Typically, the major characteristic of the reported TPs is an α-1,3-glycosidic linkage in the backbone chain with highly branched side chains [6]. Due to the complicated structures in compositions and branches, the precise chemical structures of TPs vary greatly depending on the sources, extraction methods, and purification processes [3,4].

The pace of population aging is increasing across the world. Age-related diseases constitute a huge burden on the medical systems of countries globally [7]. Accordingly, aging and anti-aging have always attracted intensive attention from researchers worldwide [8]. Aging is a complicated physiological process that results in a decrease in the function of tissues and organs. The most visible signs of aging are observed in the skin, including wrinkling, rough texture, loss of elasticity, etc. [9]. Skin is a special organ that is the interface between the body and the external environment and is the first line of defense against pathogens and dangerous challenges [10]. The skin’s structural components include several types of cells, such as fibroblasts, keratinocytes, specialized immune cells, and a high proportion of the extracellular matrix (ECM) such as collagen fibers, proteoglycans, and glycosaminoglycans [11]. Skin closely connects with the neuro-immuno-endocrine axes of the body, and the structural and functional homeostasis of the skin is vital for healthy aging [9]. Several studies have shown that the oral administration of TPs slows down skin aging in d-Galactose-induced aging mice, characterized by a decreased loss of hydroxyproline and hyaluronic acid in the skin of aged mice [12,13,14]. This improvement is supposed to be the protective effects of antioxidant enzymes related to TP treatment, including the protection of the activities of superoxide dismutase (SOD), catalase (CAT), and glutathione peroxidase (GSH-Px) in aged mice [12,13]. However, the detailed biological mechanisms of the actions of TPs on the aged skin of mammalians are still largely unknown.

Proteins are vital determinants of the physiological functions and phenotype of an organism. Proteomics is an effective tool for uncovering the whole set of proteins in an organism and illustrates the dynamic response of an organism to interventional factors that may change the degradation and/or synthesis of proteins. Over the last decade, the isobaric tags for relative and absolute quantification (iTRAQ)-based proteomics method has been extensively applied for proteomic analysis due to its advantages in precision, sensitivity, and high throughput [15,16]. In the field of exploring the bioactive mechanisms of mushroom polysaccharides, for example, 306 differential proteins were identified in colon tissues in a colitis mouse model induced by 2,4,6-trinitrobenzene sulfonic acid (TNBS) that were targeting proteins for a low dose of carboxymethyl polysaccharides from Poria cocos using the iTRAQ-based proteomics approach [17]. Based on iTRAQ proteomic analysis, *Pleurotus eryngii* polysaccharide supplementation induced a total of 113 differential proteins related to the transport and biosynthetic process in the small intestine and a total of 194 differential proteins related to the metabolic process in the colon [18].

In our previous study, three types of purified TPs with different molecular weights were prepared using the boiling water extraction method, followed by ultrasonic irradiation (US) and H_2_O_2_ treatments [19]. Of note, TPs with medium average molecular weights of 4.68 × 10^6^ Da exhibited the best performances in relieving skin aging in d-Galactose-induced aged mice among the three TPs [19]. In this study, for a better understanding of the roles of TPs in delaying skin aging, a medium-molecular-weight TP, termed NTP, was prepared according to previously described procedures [19]. After structural characterizations, the protective efficacy of NTP on skin aging in d-Galactose-induced aging mice was evaluated in terms of the content of hydroxyproline and hyaluronic acid and the activities of antioxidant enzymes in the skin. Subsequently, the iTRAQ-coupled nano-liquid chromatography–tandem mass spectrometry (n-LC-MS/MS)-based proteomics approach was applied to compare quantitative alterations in the skin proteomes between the aging mice and NTP-treated aging mice to reveal which specific proteins were involved and what the induced physiological changes were from the view of protein expressions, linked to NTP treatment in the skin. The results derived from this study may expand our understanding of the biological mechanisms regarding anti-skin aging in mammals for mushroom polysaccharides.

## 2. Results

### 2.1. Structure Characteristics of NTP

**Figure 1:**
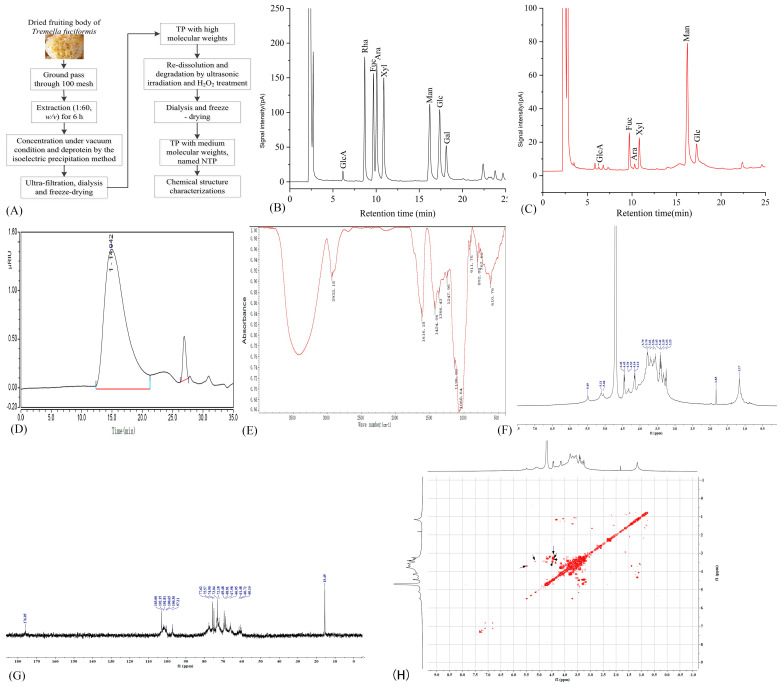
Structural characterizations of NTP. (**A**) Preparation process of NTP; (**B**) mix standards of gas chromatography (GC) spectrum; (**C**) GC spectrum of NTP; (**D**) high-performance gel permeation chromatography (HPGPC) spectrum; (**E**) FT-IR spectrum; (**F**) ^1^H NMR spectrum; (**G**) ^13^C NMR spectrum; (**H**) ^1^H-^1^H COSY NMR spectrum. GluA: glucuronic acid; Fuc: fucose; Ara: arabinose; Xyl: xylose; Man: mannose; Glu: glucose.

As presented in Figure 1B,C, the NTP is mainly composed of mannose, fucose, xylose, glucose, and arabinose at a molar ratio of 4.78:1.18:1:0.82:0.11 based on the peak area integrals, with a small amount of glucuronic acid. The profiles of the molecular weight distribution of NTP are illustrated in the HPGPC chromatogram (Figure 1C). The large peak before 20 min of retention time belonged to NTP, and the small peak at 26.9 min of retention time was attributed to the solvent. The weight-average molecular weight of NTP was 3.6 × 10^6^ Da, calculated by the curve equation of the retention time and molecular weights of the standards. The FT-IR spectrum displayed typically characteristic peaks for polysaccharide molecules, including a peak at 2932 cm^−1^ due to the stretching vibration of C-H, multiple peaks at the region of 950–1200 cm^−1^ due to the presence of C-O, C-C, and C-O-H groups, and peaks at 911 and 802 cm^−1^ attributed to the absorption of D-glucopyranosyl and α-D-mannopyranose, respectively. The ^1^H NMR spectrum of NTP exhibited signals for anomeric protons at δ 5.49, 5.11, 5.04, 4.46, 4.45, 4.34, and 4.32 ppm (Figure 1D). Based on the peak integrals of signals, a higher proportion of α-configuration and a lower proportion of β-configuration were present in the structure of molecules. The ^13^C NMR spectrum contained six anomeric carbon signals at δ 103.00, 102.15, 101.81, 100.65, 100.30, and 97.21 ppm, indicating six types of saccharide residues present in the molecules. Information from the ^1^H NMR, ^13^C NMR, and COSY NMR spectra demonstrated that NTP was mainly composed of six types of monosaccharides (Figure 1E–G), consistent with the outcomes of GC analysis. Meanwhile, a signal at δ 1.17 ppm in the ^1^H NMR spectrum was attributed to the CH_3_ moiety of fucose residue, and a signal at δ 1.83 ppm revealed the CH_2_ group, which was further manifested by the signals at δ 61.48, 60.72, and 60.14 ppm in the ^13^C NMR spectrum. The signal at δ 176.05 ppm in the ^13^C NMR spectrum demonstrated carboxyl groups present in the NTP, further confirming the presence of glucuronic acid residue. Taken together, NTP was an α-heteromannan. The structure characteristics of NTP were highly similar to those of our previously reported TPs, demonstrating the high reproducibility of the established extraction, purification, and degradation processes.

### 2.2. The Effects of NTP on the Physiological Properties of the Skin in D-Galactose-Treated Mice

**Figure 2:**
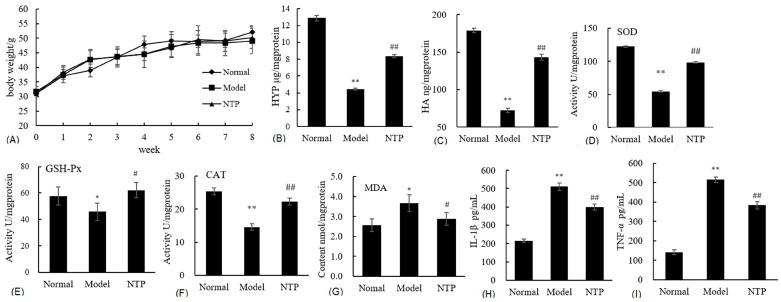
Effects of NTP on the physiological properties of the skin in galactose-treated mice. (**A**) Body weight changes; (**B**) content of hydroxyproline (HYP) in the skin; (**C**) content of hyaluronic acid in the skin; (**D**) activity of SOD; (**E**) activity of GSH-Px; (**F**) activity of CAT; (**G**) content of MDA; (**H**) content of IL-1β in the serum; (**I**) content of TNF-α in the serum. * and ** represent *p* < 0.05 and *p* < 0.01, respectively, compared with the Normal group; # and ## represent *p* < 0.05 and *p* < 0.01, respectively, compared with the Model group.

For general observations, there were no deaths in the three groups. However, the mice in the Model group were inactive, and their fur was dull and messy compared with those in the Normal group. The behavior and the color of the fur of the mice improved remarkably in the NTP group. The body weights of the mice in all groups increased slowly, and no statistically significant differences were observed between the groups during the period of the experiment (Figure 2A). To further assess the degree of skin aging, the content of hydroxyproline (HYP) and hyaluronic acid (HA) in skin tissues in the three groups, as well as the oxidative stress, was evaluated. As presented in Figs. 2B and C, the content of hydroxyproline and hyaluronic acid is much lower in the Model group than in the Normal group (*p* < 0.01), demonstrating that the eight-week d-Galactose treatments substantially accelerated skin aging in mice. Consequently, the activities of SOD, GSH-Px, and CAT significantly decreased in the Model group compared to the Normal group (*p* < 0.01), while the content of MDA was elevated considerably in the Model group compared to the Normal group (*p* < 0.05) (Figure 2D–G). Meanwhile, higher levels of inflammatory cytokine IL-1β and TNF-α were observed in the Model group in comparison with the Normal group (Figure 2H,I), suggesting the presence of severe inflammation in d-Galactose-treated mice. As a result, NTP treatment substantially alleviated the loss of hydroxyproline and hyaluronic acid and protected the activities of SOD, GSH-Px, and CAT (*p* < 0.05 or *p* < 0.01) while greatly reducing the content of MDA and the levels of inflammation with regard to IL-1β and TNF-α in d-Galactose-treated mice (*p* < 0.05 or *p* < 0.01) (Figure 2B–I). These results further confirmed the anti-skin-aging effects of NTP characterized by mitigated ECM loss, decreased oxidative stress, and reduced inflammation.

### 2.3. Mass Spectra Data Analysis and Protein Identification

**Figure 3:**
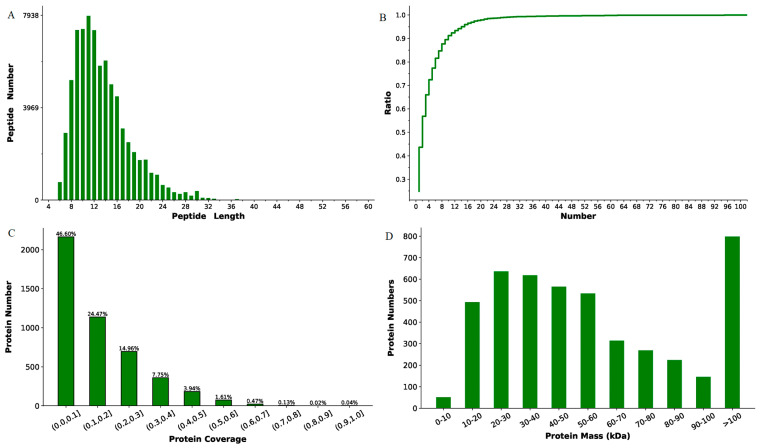
Basic proteomic data of the iTRAQ experiments. (**A**) Distribution of peptide length. (**B**) Distribution of the accumulative ratio of proteins containing unique peptides to the total identified proteins. (**C**) Distribution of protein coverage by the identified peptides. (**D**) Distribution of protein mass class among the identified proteins (in kDa).

The iTRAQ experiments generated 572,182 total spectra. A total of 35,093 peptides and 4646 proteins were identified from 76,909 matched spectra at FDR ≤ 1% (Table S1). As presented in Figure 3, the identified peptides mainly contain 7–25 amino acid residues (Figure 3A). Over 55% of the identified proteins contained no less than two unique peptides (Figure 3B). Moreover, more than half of the identified proteins showed over 10% sequence coverage (Figure 3C). Furthermore, broad ranges of protein mass distribution were observed (Figure 3D), demonstrating the wide range of proteins identified. In summary, these data indicated the high quality of the iTRAQ data and the reliability of the identified proteins.

### 2.4. Identification of the DEPs

**Figure 4:**
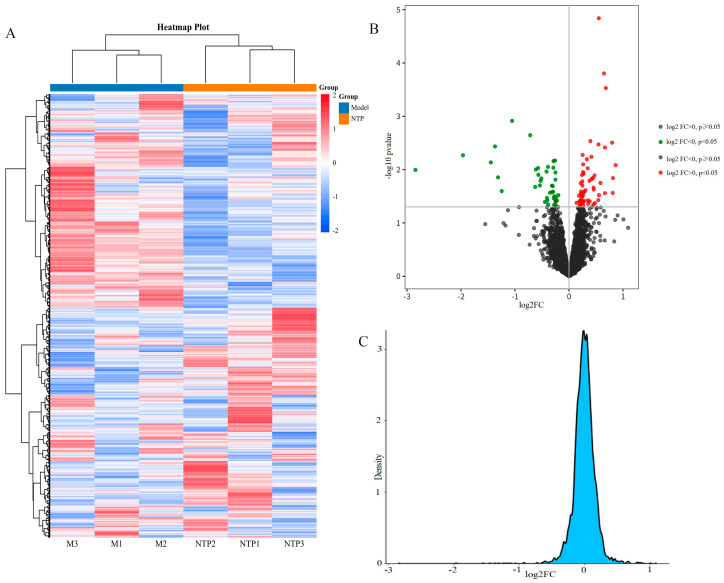
Visualization of the difference in relative abundance of the identified proteins in the skin proteomes. (**A**) Hierarchical clustering analysis of the identified proteins between samples. Abundance profiles of the identified proteins were calculated after normalization. Different colors represent different relative abundance for the identified proteins in samples. The darker the blue, the lower the expression level. Conversely, the darker the red color, the higher the expression level. Model: sample from Model group; NTP: sample from NTP group. (**B**) Volcano plots of differentially expressed proteins (DEPs) in the skin in the NTP and Model groups. Each dot represents a protein. Dots in a red color represent fold change >1 and *p* < 0.05, and dots in a green color represent fold change <1 and *p* < 0.05. (**C**) Fold-change (FC) density of DEPs. The density value represents the ratio of the number of identified proteins at that fold change to the total number of proteins.

To reflect the relationship of the expression levels of identified proteins between samples, the Euclidean distance was used for hierarchical clustering analysis. The hierarchical clustering heatmap of samples from the Model and NTP groups is shown in Figure 4A. Samples from the same group are preferentially sorted together, reflecting the highly consistent impacts of NTP on protein expressions in the skin of d-Galactose-induced aging mice. To compare and visualize differences in skin proteomes between the Model and NTP groups, a volcano plot was generated. As shown in Figure 4B, NTP treatment significantly altered the expressions of certain proteins in the skin of aging mice (dot colors in red and green). As expected, most of the identified proteins were not statistically significant in expressions, and therefore, the fold-change (FC) density exhibited a normal distribution (Figure 4C).

DEPs in the skin proteomes in the Model and NTP groups were identified using the criteria of |FC| ≥ 1.3 and *p* < 0.05. As shown in Table 1, a total of 43 proteins are obviously altered in expression, with 23 up-regulated proteins and 20 down-regulated proteins. Among them, purine nucleoside phosphorylase (1.82-fold), hexabrachion-like protein (1.76-fold), and Igh protein (1.75-fold) were the three most up-regulated proteins, while cyclin-dependent kinase 5 (0.14-fold), acetyl-coenzyme A synthetase (0.26-fold), and vesicle-fusing ATPase (0.37-fold) were the three most down-regulated proteins in response to NTP treatment.

### 2.5. GO Enrichment Analysis and KEGG Enrichment Pathway Analysis of the DEPs

**Figure 5:**
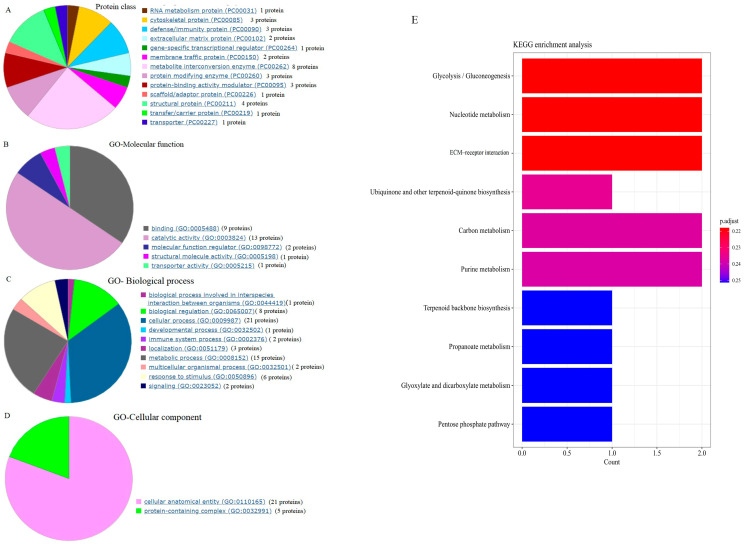
GO and KEGG enrichment analyses of the DEPs. (**A**) Protein class (33 proteins of the total 40 proteins hit); (**B**) molecular function; (**C**) biological process; (**D**) cellular component. The number of proteins with GO terms in the sub-categories of molecular function, biological process, and cellular component is shown. (**E**) KEGG pathway analysis of the DEPs (top 10 pathways are shown).

Thirty-three proteins from forty DEPs (Igh, single-chain Fv, and ribosomal protein 10 not found in the database) were classified into 13 classes by PANTHER (Figure 5A). Most DEPs belong to metabolism-related enzymes and structural components. The GO terms of the DEPs were categorized into three functional categories: “molecular function”, “biological process”, and “cellular component”. With regard to molecular function, the DEPs were mainly involved in binding and catalytic activity (Figure 5B). With regard to the biological process, 10 processes were identified. Of them, biological regulation, cellular process, metabolic process, and immune system response were the predominantly enriched biological processes (Figure 5C). With regard to cellular components, most of the DEPs only participated in the cellular anatomical entity and protein-containing complex (Figure 5D). Further, KEGG enrichment pathway analysis was performed. All of the DEPs were associated with 37 pathways (Table 2). The obviously enriched pathways were glycolysis/gluconeogenesis, nucleotide metabolism, and ECM–receptor interaction (*p* = 0.22, Figure 5E).

### 2.6. Analysis of Protein–Protein Interactions

**Figure 6:**
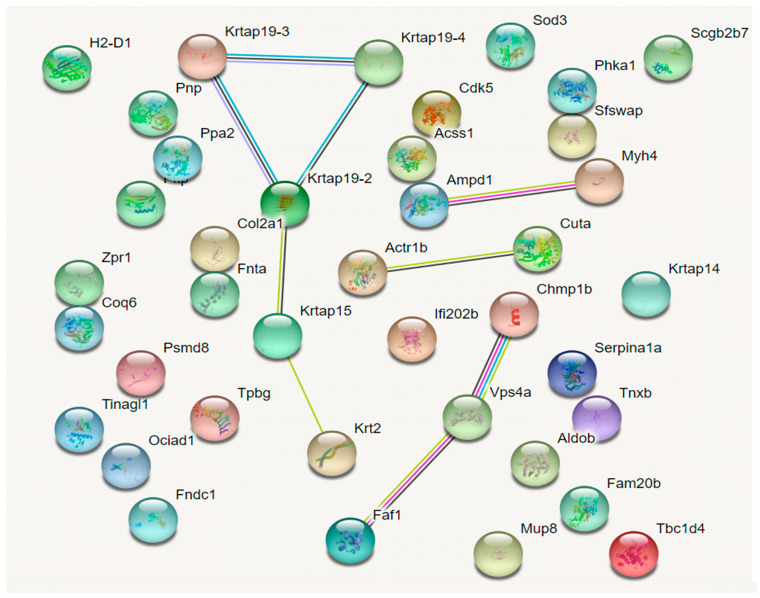
Protein–protein interaction analysis by STRING.

To better understand the biological mechanisms of NTP underlying the amelioration of skin aging, a protein–protein interaction network analysis was conducted by STRING. As shown in Figure 6, an interaction network including thirty-nine DEPs is mapped (four proteins were not found in the STRING database). A small network formed by Krtap19-2, Krtap19-3, Krtap19-4, Krtap19-4, and Krt2 and three functional modules (Ampd1-Myh4, Actr1b-Cuta, and Chmp1b-Vps4a-Faf1) were enriched. These results suggested that indirect correlations were dominant among the DEPs.

### 2.7. Validation of DEPs in mRNA Expression Levels by RT-qPCR

**Figure 7:**
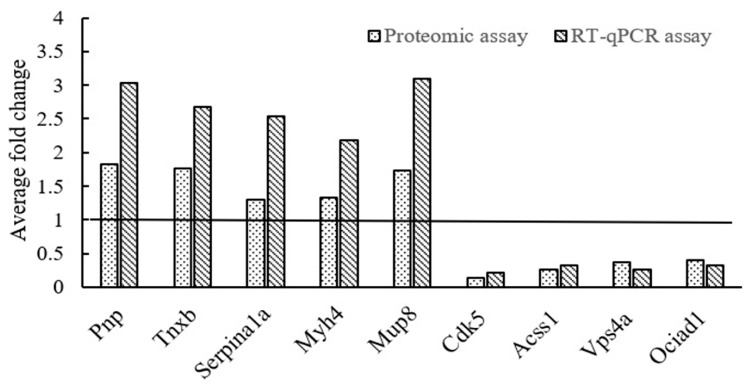
Validation of nine DEPs by RT-qPCR assay.

Based on the fold-change values of DEPs, nine genes representing nine DEPs were chosen to validate their mRNA levels by the RT-qPCR assay. As shown in Figure 7, the average fold changes of these genes are basically consistent with those of the quantitative proteomic results, illustrating the high reliability of the proteome profiles of skin tissues in d-Galactose-treated mice.

## 3. Discussion

A heteromannan extracted from the fruiting body of *T. fuciformis*, termed NTP, consisting of six monosaccharides with weight-average molecular weights of 3.6 × 10^6^ Da exhibited excellent anti-skin-aging actions on d-Galactose-induced aging mice, characterized by decreased loss in the content of hydroxyproline and hyaluronic acid, alleviated oxidative stress, and reduced inflammation. Moreover, a total of 43 DEPs were identified from 4646 proteins in response to NTP treatment in the skin proteome using the iTRAQ-based proteomics approach, of which 23 proteins were substantially up-regulated and 20 were down-regulated. These DEPs were mainly involved in cellular and metabolic regulations, immune system responses, and structural components.

d-Galactose-induced animal aging models have been widely utilized for anti-aging research in natural antioxidant exploration [20]. The excessive production of reactive oxygen species (ROS) and suppression of the antioxidant system in cells are the most feasible mechanisms for d-Galactose-induced skin aging [20]. Excessive ROS leads to oxidative DNA damage, which activates transcription factors nuclear factor-κB (NF-κB) and transcription factor activator protein-1 (AP-1) via the mitogen-activated protein kinase (MAPK) signaling pathway, resulting in the suppression of collagen production and the overexpression of matrix metalloproteinases (MMPs) in fibroblasts [21,22]. MMPs can degrade the components of the ECM, including collagen and proteoglycans [23]. Meanwhile, excessive ROS also leads to chronic inflammation, known as inflamm-aging [24]. Inflammatory cytokines such as TNF-α can also induce the secretion of MMPs [25], which is another factor that accelerates skin aging. Therefore, increased levels of degraded ECM, reduced collagen synthesis, and inflammation are pathologies in d-Galactose-induced skin aging, which are consistent with physiological changes in human aging, such as the decreased content of collagen and hyaluronic acid in the skin [26,27], increased oxidative stress [28], and chronic inflammation [29]. In the current study, NTP mitigated the loss of hydroxyproline and hyaluronic acid, reduced oxidative stress, and decreased the inflammatory level in d-Galactose-induced aging mice. Thus, it can be deduced that the ameliorations in the loss of hydroxyproline and hyaluronic acid in the skin were partially attributed to the protection of the activities of antioxidant enzymes and anti-inflammation effects induced by NTP. Naturally, macromolecular antioxidants (e.g., polyphenols, polysaccharides) protect against oxidant damage by inducing complicated intracellular signals for expressing antioxidant enzymes in vivo [30,31]. For example, a heteropolysaccharide with a molecular weight of 9.4 kDa from the mushroom *Suillellus luridus* activated antioxidant enzymes by regulating the nuclear factor erythroid 2-related factor 2 (Nrf2)/heme oxygenase-1 (HO-1) signaling pathway [32]. However, little attention has been paid to the detailed biological mechanisms of how mushroom polysaccharides regulate the expression of antioxidant enzymes in vivo since other signaling pathways, such as PI3K/Akt and NF-κB, have complex crosstalk with Nrf2-mediated pathways [33]. Previous investigations have shown that TPs exhibit anti-inflammation actions through the modulation of the gut microbiota and mucosal immune responses in mice [34,35]. Skin physiological conditions are closely associated with the gut microbiome and the function of the immune system via the gut–skin axis [36]. Gut–skin crosstalk is carried out through the activities of immunological components present between the gut and the skin, such as short-chain fatty acids (SCFAs), antimicrobial peptides, and cytokines [37]. Bioactive polysaccharides can influence intestinal functions, including intestinal barrier function, mucosal immune function, gut microbiota, and metabolites, to promote overall health [38]. Therefore, future studies are needed to uncover how TPs regulate the intracellular signals and thereby induce the expressions of antioxidant genes via the modulation of the gut community and co-metabolites in an aging mammalian model.

The DEPs induced by NTP may help to elucidate the biological mechanisms as well as the potentially beneficial effects of mushroom polysaccharides on anti-skin aging. Aging is the most important determinant of cellular dysfunction and metabolic decline for mammalian species, such as increased oxidative stress and mitochondrial decline [39]. In D-Galactose-treated mice, overloaded galactose leads to the overproduction of ROS through the mitochondrial respiratory chain, which in turn cumulatively causes mitochondrial oxidative impairments [20]. Purine nucleoside phosphorylase (PNP) and adenosine monophosphate (AMP) deaminase 1 are crucial enzymes in purine nucleotide metabolism. Uric acid, a product of purine metabolism, is a typical antioxidant [40]. The markedly up-regulated PNP and AMP deaminase 1 might reflect a high production of uric acid in the body, which could be a compensatory increase induced by NTP to compensate for the capacity of antioxidant enzymes to eliminate ROS because the oxidative stress was still higher in the NTP-treated mice compared with the normal mice. Furthermore, superoxide dismutase was also up-regulated, in accordance with an increase in the activity of SOD, further confirming the reduction in oxidative stress in NTP-treated mice. A decrease in the efficiency of oxidative phosphorylation by decreasing ATP production and altering respiratory function induced by d-Galactose was reported [20]. The DEPs related to energy production were up-regulated, including inorganic pyrophosphatase, phosphorylase kinase regulatory subunit b, and ubiquinone biosynthesis monooxygenase COQ6. Inorganic pyrophosphatase and phosphorylase kinase regulatory subunit b are involved in oxidative phosphorylation. Ubiquinone biosynthesis monooxygenase COQ6 catalyzes the biosynthesis of coenzyme Q, which is essential to energy production in the mitochondrial respiratory chain [41]. Furthermore, a down-regulation of fructose-bisphosphate aldolase observed in the current study indicated the activation of AMP-activated protein kinase (AMPK), resulting in the generation of ATP via glycolysis [42]. These results suggested that energy production increased in response to NTP treatment in d-Galactose-treated mice. Acetyl-coenzyme A synthetase catalyzes acetate to acetyl-coenzyme A (acetyl-CoA), which is fundamentally important in anabolism and energy generation, as well as in the regulation of gene expression in aging [43]. The acetyl-CoA level was found to be increased dramatically in the mouse liver during aging [44]. Therefore, the down-regulation of acetyl-coenzyme A synthetase might reflect a reduced level of acetyl-CoA in the body, which might be beneficial for anti-aging and warrants further exploration. 

Additionally, major urinary protein 5 was markedly up-regulated by 1.74-fold. In a previous study, the expression of major urinary protein 5 was down-regulated by dietary restriction and up-regulated by re-feeding in mice [45], demonstrating that it might play an important role in the metabolic process. The expression of major urinary protein 1 was found to be associated with the gut microbiota [46]. The phospholipid transfer protein regulates the transfer of a number of lipid components between lipoproteins and cells, and its abnormal expression is associated with many lipid-related metabolic diseases as well as central nervous system diseases [47]. A high activity of the phospholipid transfer protein was related to a lower LPS concentration in plasma in humans [48]. The increased expressions of major urinary protein 5 and phospholipid transfer protein in the current study might be due to the modulation of the gut microbiota of NTP since TPs can change the compositions of the gut microbiota and metabolites [34]. The detailed functions of NTP related to metabolism and the underlying mechanisms need to be further explored in future studies. 

Protein kinases modulate several metabolic pathways associated with the aging process [49]. Cyclin-dependent kinase 5 plays key roles in cellular processes such as gene regulation, cell survival, and apoptosis [50]. For example, the abnormal activation of cyclin-dependent kinase 5 induced by oxidative stress leads to neuronal apoptosis [51], suggesting the key role of cyclin-dependent kinase 5-mediated intracellular signaling in age-related brain dysfunction. Furthermore, cyclin-dependent kinase 5-mediated hyperphosphorylation promotes the development of age-related endothelial senescence and atherosclerosis [52]. Surprisingly, cyclin-dependent kinase 5 was the most down-regulated (0.14-fold) protein in our study. This might indicate the potential beneficial roles of NTP in age-related neurodegenerative diseases and vascular aging, which warrants further exploration. Taken together, the up-regulated and down-regulated proteins with differential expression involved in cellular and metabolic regulations might imply the potential effects of NTP on metabolic dysfunctions in the aging process since mushroom polysaccharides exhibit potential therapeutic benefits in the management of metabolic syndromes [53]. However, more studies are needed to elucidate the effects of TPs on anti-aging regarding metabolism and the underlying molecular mechanisms to better understand the health-promoting effects of mushroom polysaccharides.

With aging, skin immunity declines, characterized by a decrease in the number of immune cells, such as Langerhans cells, and impaired immune function, such as decreased antigen-specific immunity [11]. In the present study, d-Galactose treatment induced a high level of inflammation in mice, which was consistent with previous results [54]. Among the DEPs, the Igh protein, immunoglobulin heavy variable 5-16, and single-chain FV belong to the components of antibodies. H-2 class I histocompatibility antigen participates in antigen processing. Alpha-1-antitrypsin 1-1 is a serine protease inhibitor in the circulation system and possesses many other biological functions, including modulating immunity, inflammation, and proteostasis [55]. Interestingly, a decrease in the activity of PNP in spleen lymphocytes in aged mice was observed [56]. Additionally, an increase in adenosine deaminase and PNP activities was also associated with both humoral and cellular immune responses for T and B lymphocytes in immunized mice [57]. All but one (Ifi202b) DEPs associated with the biological functions of the immune response were overexpressed, indicating increased skin immunity after NTP treatment in aging mice, as TPs exhibit outstanding immune-modulating activity [6]. Ifi202b is a response to interferon-beta activation. Recently, studies reported that the overexpression of Ifi202b caused obesity in mice [58], suggesting the other beneficial role of down-regulating Ifi202b in NTP-treated aging mice. Further studies are needed to explore the potential roles of the down-regulation of Ifi202b in an aging mammalian model.

A reduced amount of ECM occurs in the aging process [21]. Collagen alpha-1(II) chain, fibronectin type III domain-containing 1, and tubulointerstitial nephritis antigen-like 1 protein are components of the ECM. Tubulointerstitial nephritis antigen-like 1 protein interacting with other ECM components such as collagen and fibronectin plays a role in cell adhesion and interaction [59]. In addition to providing support to cells, the ECM also regulates intercellular signals [60]. The up-regulation of the three DEPs might be related to ECM remodeling associated with anti-aging effects on the skin [21], as an increase in the contents of hydroxyproline and hyaluronic acid was observed in aging mice in response to NTP treatment. Myosin 4 is a member of the Myosin family. Beta-centractin belongs to actin-related proteins. Myosin–actin interactions play key roles in the delivery of intracellular substances within cells for maintaining the normal function of cells [61]. Myosin content decreases with aging [62]. Beta-centractin and Myosin 4 were up-regulated in the present study, which might be associated with improved cell function in aging skin. Keratins provide support and barrier function in epithelial cells on the surface of the skin. In the current study, six DEPs related to keratins were identified. Of them, five proteins were markedly down-regulated. These were consistent with our previous observations that the majority of DEPs related to keratins were down-regulated by *Lentinula edodes*-derived polysaccharides in the intestinal tissues in mice [63], suggesting a gut-microbiota-involved role of mushroom polysaccharides in the modulation of protein expressions related to cellular structural components. However, few studies are available on the biological mechanisms of *Tremella* polysaccharides in modulating the expression of these DEPs to date. Furthermore, the functional relevance of cytoskeleton-associated protein expressions in anti-aging is still unclear. Therefore, further studies are needed to investigate these interesting topics to facilitate the understanding of the anti-aging roles of mushroom polysaccharides.

Of note, some DEPs, such as cyclin-dependent kinase 5 (CDK5, 0.14-fold), acetyl-coenzyme A synthetase (acetyl-CoA synthetase, 0.26-fold), and purine nucleoside phosphorylase (PNP, 1.82-fold), were substantially regulated by NTP. Emerging evidence indicates the role of the gut microbiota in the regulation of DEPs. For example, honokiol has been shown to suppress the CDK5-involved signaling pathway to ameliorate cognitive deficits in the Alzheimer’s disease mouse model (TgCRND8 mouse) via the modulation of the gut microbiota [64]. Further fecal microbiota transplantation from normal mice confirmed the role of the gut microbiota in alleviating cognitive deficits by suppressing the activation of the CDK5-involved pathway in TgCRND8 mice [65]. Acetyl-CoA synthetase activity is finely regulated by cyclic AMP (cAMP) [66]. Gut microbiota-derived metabolites could activate the cAMP/PKA/NF-κB pathway to alleviate ulcerative colitis in mice [67]. PNP is a critical enzyme in purine metabolism. Herbal-based interventions have emerged as valuable approaches in the regulation of host purine homeostasis through the modulation of the gut microbiota [68]. However, few studies have been reported on the detailed molecular mechanism by which the gut microbiota regulates these DEPs. As a reasonable deduction, the DEPs identified in the present study are highly likely to be regulated by NTP via the modulation of the gut microbiota because TPs have shown the capacity to change the gut microbiota in mice [69]. Accordingly, more studies using gene intervention technology and germ-free animal models are necessary to reveal how NTP affects the expression and activity of these DEPs via the regulation of the gut microbiota for a better understanding of the molecular mechanisms of NTP in anti-aging, which can facilitate the development of new strategies for the prevention and management of aging-related diseases.

Also, there were some limitations in our study. First, given the multiple hallmarks of skin aging [70], the detected indicators were relatively few, and the skin aging status of the d-Galactose-treated mice could not be comprehensively evaluated. Second, although the d-Galactose-induced aging model is widely used for anti-aging therapeutic intervention studies, d-Galactose-induced aging signatures are not completely consistent with naturally occurring aging characteristics in rodents [70]. Recent investigations across different genetic backgrounds have uncovered metabolic phenotypic diversity in mice [71]. Aging and metabolism are inextricably associated. To fully understand the anti-aging roles of NTP, further studies using different metabolic phenotypes and genetic background models will overcome the shortcomings of the current findings. Third, the dose–response relationship of NTP in anti-skin aging was overlooked, with the aim of assessing the potential benefits and the underlying mechanisms of NTP regarding skin aging protection for human consumption at an achievable dose in real-life applications. Thus, in order to better understand the dose–response relationship of NTP and promote its application, it is necessary to investigate the protective effects of different doses of NTP in future studies.

Currently, the skin proteome profiles provide an overview on the response to NTP treatment in aged mice. However, there are huge gaps between the biological significance of these DEPs and skin aging. Meanwhile, the biological mechanisms by which mushroom polysaccharides regulate the DEPs are largely unknown. Therefore, in future work, well-designed experiments using gene knock-out or knock-down mammalian models, as well as sterile animals, are necessary to uncover the roles of the critical DEPs in cutaneous aging and the underlying biological mechanisms. Moreover, increasing research has documented that mushroom polysaccharides have excellent antioxidant potential [72,73,74]. Mushroom components (e.g., polysaccharides) have been applied in aesthetic medicine for external (creams, lotions) and internal (dietary supplements) uses for the treatment and management of cutaneous symptoms for a few decades [75]. As the population ages, the need to promote healthy aging grows. Dietary interventions and nutritional supplementation are recognized as effective ways to promote healthy aging among the elderly [76]. However, most investigations supporting the anti-aging properties of mushroom polysaccharides have been carried out using in vivo or in vitro models, with limited clinical trials [74]. Therefore, clinical trials or nutritional investigations of TPs or NTP performed on older people are urgently required to explore and validate the potential benefits of TPs in real-life anti-aging applications.

## 4. Materials and Methods

### 4.1. Material Preparation

The dried fruiting body of *T. fuciformis* was purchased from Gutian, Fujian province, China. The powder of *T. fuciformis* was obtained after being crushed and passed through a 40-mesh sieve. NTP was prepared using the previously established protocol (Figure 1A) [19]. In brief, approximately 5 g of TPs with high molecular weights was obtained from 14 g of dried fruiting bodies of *T. fuciformis* after extraction, purification, and freeze-drying. Then, the obtained TP was degraded by US and H_2_O_2_ treatment, and the viscosity of the TP solution was 110.7 mPa·S at the beginning and 20.5 mPa·S at the end of the treatment. The resulting solution was dialyzed (cut-off 1,000 Da) and lyophilized to obtain NTP. The yield of NTP was 32.7% of the dry weights of the fruiting bodies of *T. fuciformis*. The carbohydrate content of NTP was 93.6% (dry weight), determined by the phenol–sulfuric acid method with mannose as the standard, and its protein content was 0.5% detected by the bicinchoninic acid (BCA) method [77]. The content of uronic acids of NTP was 6.42%, as determined by the sulfamate/m-hydroxydiphenyl method using glucuronic acid as the standard [78].

### 4.2. Structure Characterizations

The monosaccharide composition analysis of NTP was conducted using the acetylation derivatization method [19]. The molecular weight distributions of NTP were determined by high-performance gel permeation chromatography (HPGPC) using a gel permeation chromatography system (Ultimate 3000, Thermo Fisher Scientific Inc., Waltham, MA, USA) [79]. Fourier transform infrared (FT-IR) spectroscopy was conducted using an FT-IR spectrometer (Nicolet IS50-Nicolet Continuum, Thermo Fisher Scientific Inc., Waltham, MA, USA) [13]. The ^1^H and ^13^C nuclear magnetic resonance (NMR) spectra of NTP, including one-dimensional ^1^H and ^13^C and two-dimensional ^1^H-^1^H correlation spectroscopy (COSY), were obtained on a 600 MHz NMR spectrometer (Bruker AVANCE III, Bruker BioSpin Group, Billerica, MA, USA) at 298 K [19].

### 4.3. Animal Experiment

Four-week-old male specific-pathogen-free (SPF)-grade Kunming mice (body weight 18±2 g) were purchased from Guangdong Medical Experimental Animal Center. The animal experiment was approved by the Ethics Committee of Guangzhou University of Chinese Medicine (approval No. 20200620001) and carried out at the Laboratory Animals Center at Guangzhou University of Chinese Medicine, Guangzhou, China. The mice had free access to food and water in an SPF laboratory environment: room temperature 22 ± 2 °C under 60–80% RH with a 12 h light/dark cycle. Mice were acclimatized for two weeks before the formal experiment. Experimental procedures were performed following the Health Guide for the Care and Use of Laboratory Animals of National Institutes and the Chinese legislation on the use and care of laboratory animals.

A total of 24 male mice were randomly divided into three groups: Normal group (oral administration of physiological saline, n = 8), Model group (oral administration of physiological saline, n = 8), and NTP group (oral administration of NTP with a dosage of 100 mg/kg of body weight (BW) per day, n = 8). Mice in the Normal group were intraperitoneally injected daily with physiological saline, and those in the remaining groups were intraperitoneally injected daily with d-Galactose (Shanghai Macklin, Shanghai, China) at a dosage of 120 mg/kg of BW [19,80]. The treatment lasted for eight weeks. The body weight of mice was measured at the beginning of the treatment and once a week during the experiment. The daily activities, aging signs, and food intake of the mice were recorded. At the end of the experiment, blood samples were collected after anesthesia, and then mice were euthanized. A 1 × 1 cm^2^ sample of dehaired dorsal skin was collected from each mouse and pre-frozen with liquid nitrogen. All samples were stored at –80 ° C immediately until further analysis within two months.

### 4.4. Measurement of the Content of Hydroxyproline, Hyaluronic Acid, and MDA and the Antioxidant Enzyme Activities in the Skin Tissues

The skin sample was cut into pieces, ground, and defatted according to previous procedures [19]. Subsequently, 10% skin tissue phosphate-buffered saline (PBS) homogenates were prepared, and the supernatants were obtained by centrifugation (5000× *g* for 15 min at 4 °C). After protein quantification in the supernatants using a BCA kit (CoWin Biosciences, Beijing, China), the content of hydroxyproline and hyaluronic acid in the supernatants was determined using kits according to the manufacturer’s instructions (Jiangsu Meimian Biotech., Yancheng, China). Meanwhile, the activities of SOD, GSH-Px, and CAT and the content of MDA in the supernatants were determined according to the kits’ instructions (Nanjing Jiancheng Bioengineering, Nanjing, China).

### 4.5. Measurement of IL-1β and TNF-α in Serum

The content of IL-1β and TNF-α in the serum was determined according to the ELISA kit instructions of the manufacturer (Jiangsu Meimian Biotech., Yancheng, China).

### 4.6. Protein Extraction

Three skin samples representing three biological replicates were randomly selected from each group (Model and NTP groups) for iTRAQ proteomic analysis. Proteins were extracted as per the previously reported method [81]. In brief, the sample was ground into powder in liquid nitrogen, followed by extraction in protein lysis buffer (100 mM ammonium bicarbonate, 8 M urea, pH = 8). The suspension was shaken, mixed, and sonicated (JY92-11N, SCIENTZ Ningbo, China) in an ice-water bath for 5 min to lyse fully. The supernatant was obtained by centrifugation at 12,000 *g* for 15 min at 4 °C and supplemented with 10 mM DTT (D9163, Sigma-Aldrich, St. Louis, MO, USA) to react at 56 ° C for 1 h. Iodoacetamide (IAM) (Sigma-Aldrich, St. Louis, MO, USA) was then added to react at room temperature for 1 h in the dark. Subsequently, 4 times the volume of −20 °C pre-cooled acetone was added to the reaction solution to precipitate at −20 °C for at least 2 h. The precipitate was collected by centrifugation at 12,000 *g* for 15 min at 4 °C. In total, 1 mL of −20 °C pre-cooled acetone was used to resuspend and wash the precipitate, which was centrifuged at 12,000 *g* for 15 min at 4 °C. After air-drying, an appropriate amount of dissolving solution (8 M urea, 100 mM triethylammonium bicarbonate (TEAB), pH = 8.5) was used to dissolve the precipitate. A Bradford protein quantification kit (Beyotime, Shanghai, China) was used to quantify the protein concentration, and SDS-PAGE was used to measure the quality of the protein sample. Finally, 6 protein samples were obtained from skin tissues.

### 4.7. Protein Digestion, iTRAQ Labeling, and Fractionation of Peptides

About 100 µg proteins of protein solution (8 M urea, 100 mM TEAB, pH = 8.5) were digested with trypsin (V5280, Promega, MI, USA) at a ratio of 40:1 at 37 °C overnight. After digestion, formic acid was used to adjust the pH to <3. The resulting solution was mixed and centrifuged at 12,000× *g* for 5 min at room temperature. A C18-column (Phenomenex, Torrance, CA, USA) was used to desalt the supernatant. The filtrate was then collected and lyophilized to obtain peptide samples. 

The peptide samples were redissolved in 1M TEAB buffer. An iTRAQ 8-plex kit (Sigma-Aldrich, Framingham, MA, USA) was used to label the peptides according to the manufacturer’s instructions. Peptide samples from the Model group, named M1, M2, and M3, were labeled with isobaric tags 115, 116, and 117, respectively. Peptide samples from the NTP group, named NTP1, NTP2, and NTP3, were labeled with isobaric tags 118, 119, and 121, respectively. The labeling process was conducted at room temperature for 2 h. After terminating the reaction with 100 μL of 50 mM Tris-HCl (pH = 8), the labeled peptides were mixed in an equivalent ratio, desalted, and then freeze-dried.

To reduce the complexity of the peptides, the labeled peptide mixtures were separated by high-pH reversed-phase (RP) separation according to the previously described method with some modifications [82]. Briefly, the labeled mixtures were dissolved in mobile phase A (2% acetonitrile, 98% water, ammonia water adjusted to pH = 10) and separated on a RIGOL L-3000 HPLC system (RIGOL Technologies, Beijing, China) equipped with an X Bridge BEH C18-column (Waters, 4.6 × 250 mm, 5 μm bead size). The elution parameters were set as follows: flow rate 1 mL/min; column temperature 45 °C; buffer A starting from 97% to 95% for min 0–10, from 95% to 80% for min 10–30, from 80% to 60% for min 30–48, from 60% to 50% for min 48–50, and from 50% to 30% for min 50–53; and mobile phase B (98% acetonitrile, 2% water) from 70% to 100% for min 53–54. The elution solvent was collected every minute. The entire elution process was monitored at 214 nm absorbance. Finally, ten combined fractions were obtained according to the chromatography profiles of peptides and then freeze-dried.

### 4.8. LC-MS/MS Analysis

The labeled peptide fractions were separated as previously described with slight modifications [83]. Each fraction was redissolved in mobile phase A (100% water, 0.1% formic acid). The fraction separation was performed using an EASY-nLC 1200 nanoUPLC (Thermo Fisher Scientific Inc., Waltham, MA, USA) system equipped with a pre-column (4.5 cm × 75 μm, 3 μm-RP C18) and analytic column (15 cm × 150 μm, 1.9 μm-RP C18, Thermo Scientific). The fraction of peptides was eluted through a gradient procedure at a flow rate of 600 nL/min: mobile phase A starting from 94% to 88% for min 0–2, from 88% to 65% for min 2–49.5, and from 65% to 50% for min 49.5–51.5 and mobile phase B (80% acetonitrile, 0.1% formic acid) from 50% to 100% for min 51.5–52.5, finally 100% for min 52.5–60. Eluted peptides were directly analyzed with a Q-Exactive Mass Spectrometer (Thermo Fisher Scientific, Waltham, MA, USA). The parameters of the mass spectrometer were set as follows: data-dependent positive-ion mode with a mass range of 407–1500 *m/z*; the resolution of the primary MS was 60000 (200 *m/z*); the maximum capacity of the C-trap was 3×10^6^; and the maximum injection time was 20 ms. The parent ions with the top 40 ion strengths were selected for subsequent MS/MS analysis. The resolution of MS/MS was set at 1500 (200 *m/z*) with the collision energy set at 30 eV. The maximum capacity of the C-trap was set at 5 × 10^4^, the maximum injection time was 45 ms, and the dynamic exclusion duration was 20 s. Raw data of MS were generated for further analysis.

### 4.9. iTRAQ Data Analysis

Raw data were imported into Proteome Discoverer 2.2 software (Thermo, Waltham, MA, USA) for peptide searching against the UniProt database (81611 sequences). The parameters were set as follows: enzyme: trypsin; maximum missed cleavage sites: 2; precursor mass tolerance: 10 ppm; fragment mass tolerance: 0.02 Da; dynamic modification: oxidation/+15.995 Da and iTRAQ 8 plex/+304.205Da; N-terminal modification: acetyl/+42.011Da and iTRAQ 8plex/+304.205Da; and static modification: Carbamidomethyl/+57.021Da. To improve the quality of protein identification, Proteome Discoverer 2.2 was used to further filter the search results: peptide spectrum matches (PSMs) with a reliability of ≥99% were considered credible PSMs, and proteins that contain at least one unique peptide were regarded as credible proteins. Additionally, false discovery rate (FDR) validation was performed to remove peptides and proteins with an FDR value greater than 1%. For protein quantitative comparison, proteins with fold changes (FCs) ≥ 1.3 or ≤0.77 and a *p* value < 0.05 based on a *t*-test were considered differentially expressed proteins (DEPs) [84].

### 4.10. Bioinformatics Analysis

The hierarchical clustering analysis of the identified proteins was performed by Cluster 3.1 based on column-mean-centered expression levels and Euclidean distance. Functional annotations and Gene Ontology (GO) analysis of the DEPs were performed using PANTHER version 16.0 (Protein Analysis THrough Evolutionary Relationships) (www.pantherdb.org) [85]. The Kyoto Encyclopedia of Genes and Genomes (KEGG) enrichment pathway analysis of DEPs was conducted on an online platform based on the KEGG database (hiplot-academic.com) [86]. Protein–protein interactions were analyzed using version 11.5 of STRING (Search Tool for the Retrieval of Interacting Genes/Proteins) (string-db.org) [87].

### 4.11. Validation of Proteomic Profiles by Real-Time Quantitative Reverse Transcription PCR (RT- qPCR) Analysis

Skin tissues were ground into powder under liquid nitrogen, and the total RNA was extracted using TRIzol reagent (Invitrogen, Thermo Fisher Scientific Inc., Carlsbad, CA, USA). Then, reverse transcription to cDNA was carried out using a HiFiScript gDNA removal cDNA synthesis kit according to the manufacturer’s instructions (CWBio, Taizhou, China). Nine genes corresponding to nine representative DEPs were chosen for RT-qPCR validation. The primer sequences of the 9 genes are listed in Table 3. The RT-qPCR assay was conducted on an ABI 7500 Sequence Detection System (Applied Biosystems, Carlsbad, CA, USA) using a SYBR qPCR Mix kit (Thermo Fisher Scientific Inc., Carlsbad, CA, USA). The parameters of amplification were set as follows: initial denaturation temperature: 95 °C for 200 s; cycles: 40; denaturation temperature: 95 °C for 15 s; annealing temperature: 60 °C for 15 s; and extension temperature: 72 °C for 20 s. Glyceraldehyde-3-phosphate dehydrogenase (GAPDH) was selected as a control. The relative expression level of validated genes was normalized to the level of the control gene, and fold change was calculated using the method of 2^−ΔΔCt^ [88]. Three biological replicates were measured from the Model and NTP groups, respectively.

### 4.12. Statistical Analysis

Data are expressed as mean ± SD. The one-way ANOVA test, together with Tukey’s test, was applied for statistical analysis to identify the differences among groups using SPSS 22.0 (IBM, Armonk, NY, USA). A *p* value less than 0.05 was considered statistically significant.

## 5. Conclusions

In this study, a heteromannan predominantly connected with α-glycosidic linkages mainly composed of mannose, fucose, xylose, glucose, and arabinose at a molar ratio of 4.78:1.18:1:0.82:0.11 also containing a low proportion of glucuronic acid with average molecular weights of 3.6 × 10^6^ Da, named NTP, was prepared from the fruiting body of *T. fuciformis*. Gavaged NTP at a dose of 100 mg/day considerably mitigated skin aging characterized by the reduced loss of hydroxyproline and hyaluronic acid; reduced oxidative stress, as evidenced by the increased activities of antioxidant enzymes and decreased levels of MDA; and decreased inflammatory levels, as evidenced by the decreased levels of IL-1β and TNF-α in d-Galactose-induced aging mice. Further, a total of 43 DEPs were identified from the skin proteome using an iTRAQ-based proteomics approach in response to NTP treatment, of which 23 were up-regulated and 20 were down-regulated. Most DEPs belong to metabolism-related enzymes and cellular structural components. GO analysis demonstrated that a high proportion of DEPs were mainly involved in the biological functions of cellular and metabolic regulations and immune system responses. Meanwhile, the glycolysis/gluconeogenesis, nucleotide metabolism, and ECM–receptor interaction pathways were obviously enriched in KEGG enrichment analysis. These results illustrate the potential biological mechanisms of NTP underlying the anti-aging effects, particularly in anti-skin aging. The findings provide new insights into the potential roles of mushroom polysaccharides in the anti-aging field. 

## Figures and Tables

**Table 1:** DEPs identified in the skin of aging mice fed with NTP.

Accession	Protein Name	Gene Name	Coverage ^a^ %	Peptides ^b^	Score ^c^	Fold Change ^d^	*p* Value
**Immune system response**							
G3UZP7	H-2 class I histocompatibility antigen	H2-D1	24	8	53.36	1.47	1.4 × 10^−5^
Q6PIP8	Igh protein	Igh	25	8	196.85	1.75	0.027
A0A0B4J1P4	Immunoglobulin heavy variable 5–16	Ighv5-16	23	2	26.73	1.59	0.0038
L0HCN1	Ifi202b	Ifi202b	4	2	1.81	0.65	0.010
A0A679F8Q6	Single-chain Fv	scFv-A36	5	1	2.97	1.59	0.028
A0A0A0MQA3	Alpha-1-antitrypsin 1-1	Serpina1a	25	9	232.24	1.3	0.016
**Cellular and metabolic regulation**							
Q91VM9	Inorganic pyrophosphatase 2	Ppa2	12	3	10.09	1.32	0.0029
Q69Z91	Acetyl-coenzyme A synthetase	Acss1	3	2	1.72	0.26	0.0054
Q3TDX2	Vesicle-fusing ATPase	Vps4a	5	2	8.77	0.37	0.0073
Q8R1S0	Ubiquinone biosynthesis monooxygenase COQ6	Coq6	5	2	2.52	1.37	0.022
A2AI87	Phosphorylase kinase regulatory subunit b	Phka1	4	5	4.36	1.34	0.015
Q3TJ66	Fructose-bisphosphate aldolase	Aldob	19	4	53.11	0.69	0.016
P23492	Purine nucleoside phosphorylase	Pnp	44	9	63.44	1.82	0.0082
Q3V1D3	AMP deaminase 1	Ampd1	7	4	11.45	1.3	0.039
Q9CX56	26S proteasome non-ATPase regulatory subunit 8	Psmd8	14	5	12.24	0.75	0.034
P49615	Cyclin-dependent kinase 5	Cdk5	11	3	4.43	0.14	0.010
Q8VCS3	Glycosaminoglycan xylosylkinase	Fam20b	5	1	2.16	0.42	0.025
O88592	Superoxide dismutase	Sod3	16	2	49.81	1.36	0.014
Q8BYJ6	TBC1 domain family member 4	Tbc1d4	2	2	1.27	1.46	0.037
Q541Z2	Farnesyltransferase	Fnta	19	6	12.42	0.76	0.038
Q9Z0L0	Trophoblast glycoprotein	Tpbg	6	2	6.13	0.61	0.0023
A2AKN8	Major urinary protein 5	Mup8	56	8	83.46	1.74	0.0031
Q3TJ28	Charged multivesicular body protein 1B	Chmp1b	8	2	1.88	1.46	0.0034
Q3TIH8	Zinc finger protein 259	Zpr1	7	3	2.15	0.7	0.015
Q3USH5	Splicing factor, suppressor of white-apricot homolog	Q3USH5	2	2	2.00	0.73	0.040
Q3UFS5	Phospholipid transfer protein	Pltp	19	7	25.35	1.39	0.042
P54731	FAS-associated factor 1	Faf1	2	1	1.05	0.65	0.021
Q9CRD0	OCIA domain-containing protein 1	Ociad1	7	1	2.70	0.4	0.014
**Structural component**							
Q5SX39	Myosin-4	Myh4	32	64	1435.55	1.33	0.0057
Q3TAQ2	Hexabrachion-like protein	Tnxb	26	27	202.12	1.76	0.014
Q9CQ89	Protein cut A	Cuta	8	1	13.92	0.39	0.0037
Q925H6	Keratin-associated protein 19-3	Krtap19-3	16	1	19.39	0.76	0.0089
O08640	Keratin-associated protein 14	Krtap14	21	2	14.22	0.67	0.0093
Q925I0	Keratin-associated protein 19-2	Krtap19-2	9	1	8.21	0.68	0.013
B2RTP7	Krt2 protein	Krt2	3	2	12.69	1.5	0.018
Q925H7	Keratin-associated protein 19-4	Krtap19-4	17	1	2.52	0.69	0.020
Q8R5C5	Beta-centractin	Actr1b	28	7	29.52	1.37	0.024
A2AM97	Ribosomal protein 10	RP23-436K3.4-001	30	9	49.94	0.75	0.011
P28481	Collagen alpha-1(II) chain	Col2a1	5	5	40.46	1.39	0.013
E9Q043	Fibronectin type III domain-containing 1	Fndc1	3	3	8.86	1.49	0.030
Q9QZU5	Keratin-associated protein 15-1	Krtap15-1	38	3	35.46	0.76	0.045
D3YYY1	Secretoglobin	Scgb2b7	7	1	2.18	1.57	0.00016
Q99JR5	Bulointerstitial nephritis antigen-like protein	Tinagl1	9	3	10.44	1.37	0.045

^a^, the coverage of protein sequence. ^b^, peptide number matched a protein. ^c^, score for protein matching; the higher the score, the higher the confidence. ^d^, the ratio of the average relative expression level of the NTP group to the Model group. *p* value, *t*-test.

**Table 2:** KEGG enrichment pathway analysis of the DEPs induced by NTP.

ID	Description	Gene Ratio	Bg Ratio	*p* Value	*p* Adjusted	q Value	Gene ID	Count
mmu00010	Glycolysis/gluconeogenesis	2/21	67/8979	0.0105	0.2179	0.1922	Aldob/Acss1	2
mmu01232	Nucleotide metabolism	2/21	84/8979	0.0162	0.2179	0.1922	Ampd1/Pnp	2
mmu04512	ECM–receptor interaction	2/21	88/8979	0.0177	0.2179	0.1922	Tnxb/Col2a1	2
mmu00130	Ubiquinone and other terpenoid-quinone biosynthesis	1/21	11/8979	0.0254	0.2353	0.2075	Coq6	1
mmu01200	Carbon metabolism	2/21	121/8979	0.0320	0.2368	0.2088	Aldob/Acss1	2
mmu00230	Purine metabolism	2/21	134/8979	0.0386	0.2379	0.2098	Ampd1/Pnp	2
mmu00900	Terpenoid backbone biosynthesis	1/21	23/8979	0.0525	0.2513	0.2216	Fnta	1
mmu00640	Propanoate metabolism	1/21	31/8979	0.0701	0.2513	0.2216	Acss1	1
mmu00630	Glyoxylate and dicarboxylate metabolism	1/21	32/8979	0.0723	0.2513	0.2216	Acss1	1
mmu00030	Pentose phosphate pathway	1/21	33/8979	0.0745	0.2513	0.2216	Aldob	1
mmu00051	Fructose and mannose metabolism	1/21	36/8979	0.0810	0.2513	0.2216	Aldob	1
mmu00760	Nicotinate and nicotinamide metabolism	1/21	41/8979	0.0917	0.2513	0.2216	Pnp	1
mmu00620	Pyruvate metabolism	1/21	44/8979	0.0981	0.2513	0.2216	Acss1	1
mmu03050	Proteasome	1/21	47/8979	0.1045	0.2513	0.2216	Psmd8	1
mmu05030	Cocaine addiction	1/21	48/8979	0.1066	0.2513	0.2216	Cdk5	1
mmu04979	Cholesterol metabolism	1/21	49/8979	0.1087	0.2513	0.2216	Pltp	1
mmu03250	Viral life cycle—HIV-1	1/21	61/8979	0.1335	0.2545	0.2244	Vps4a	1
mmu04623	Cytosolic DNA-sensing pathway	1/21	63/8979	0.1376	0.2545	0.2244	Ifi202b	1
mmu05330	Allograft rejection	1/21	63/8979	0.1376	0.2545	0.2244	H2-D1	1
mmu05332	Graft-versus-host disease	1/21	63/8979	0.1376	0.2545	0.2244	H2-D1	1
mmu04940	Type I diabetes mellitus	1/21	70/8979	0.1517	0.2673	0.2357	H2-D1	1
mmu01230	Biosynthesis of amino acids	1/21	79/8979	0.1695	0.2693	0.2375	Aldob	1
mmu05320	Autoimmune thyroid disease	1/21	79/8979	0.1696	0.2693	0.2375	H2-D1	1
mmu05416	Viral myocarditis	1/21	88/8979	0.1870	0.2693	0.2375	H2-D1	1
mmu03320	PPAR signaling pathway	1/21	89/8979	0.1889	0.2693	0.2375	Pltp	1
mmu04612	Antigen processing and presentation	1/21	90/8979	0.1909	0.2693	0.2375	H2-D1	1
mmu04610	Complement and coagulation cascades	1/21	93/8979	0.1966	0.2693	0.2375	Serpina1a	1
mmu04922	Glucagon signaling pathway	1/21	104/8979	0.2172	0.2782	0.2453	Phka1	1
mmu04974	Protein digestion and absorption	1/21	108/8979	0.2246	0.2782	0.2453	Col2a1	1
mmu04931	Insulin resistance	1/21	110/8979	0.2283	0.2782	0.2453	Tbc1d4	1
mmu04066	HIF-1 signaling pathway	1/21	114/8979	0.2356	0.2782	0.2453	Aldob	1
mmu04919	Thyroid hormone signaling pathway	1/21	120/8979	0.2464	0.2782	0.2453	Tbc1d4	1
mmu04650	Natural killer cell-mediated cytotoxicity	1/21	121/8979	0.2482	0.2782	0.2453	H2-D1	1
mmu00190	Oxidative phosphorylation	1/21	135/8979	0.2728	0.2909	0.2565	Ppa2	1
mmu04910	Insulin signaling pathway	1/21	139/8979	0.2796	0.2909	0.2565	Phka1	1
mmu05017	Spinocerebellar ataxia	1/21	141/8979	0.2831	0.2909	0.2565	Psmd8	1
mmu01240	Biosynthesis of cofactors	1/21	152/8979	0.3016	0.3015	0.2659	Coq6	1

**Table 3:** Sequence of the primers.

Gene Name	Forward Primer (5’-3’)	Reverse Primer (5’-3’)
Pnp	AGAGAGTCGTCTGCTAAAGATG	GGAGACAAACCTTTCCAATGTC
Tnxb	TGACTGGTGTGACTCAAAACTC	CCGTAGAGTAGCAGCTTATACC
Serpina1a	GACACTCACACGCAGATCCTAGAG	GGAGGAGGTGTTGGAAGGACTTG
Myh4	GGCAAACAAGCATTTACACAAC	ATCCGTCTCATATTTCGTCCTC
Mup8	CCTGAGCCTCCAGTGTTGAGTG	GGGATGCTGTATGGATAGGAAGGG
Cdk5	CAATGTACCCAGCTACAACATC	CTTCAATAGGTTCTGCAACAGG
Acss1	CAGGCAGGCTATCTACTGTATG	AACTGGTTGATCTTTAGCCTCT
Vps4a	ATTCAGCCATCAGGAGGAGGTTTG	AGCATCTGTGAGGTTGTGAGGTG
Ociad1	CTGCCACAAGTATGCTGATTAC	CTTCTTGAACTTCTCTTGGCAC
Gapdh	AGAAGGTGGTGAAGCAGGCATC	CGAAGGTGGAAGAGTGGGAGTTG

## Data Availability

Data will be made available upon request.

## Supplementary Materials

The following supporting information can be downloaded at https://www.mdpi.com/article/10.3390/molecules29215191/s1, Table S1 Identified peptides and proteins.
